# In Silico Study for Algerian Essential Oils as Antimicrobial Agents against Multidrug-Resistant Bacteria Isolated from Pus Samples

**DOI:** 10.3390/antibiotics11101317

**Published:** 2022-09-27

**Authors:** Abdelhakim Aouf, Sarah Bouaouina, Mohamed A. Abdelgawad, Mohammed A. S. Abourehab, Amr Farouk

**Affiliations:** 1 Laboratory of Applied Microbiology, Faculty of Life Sciences and Nature, University of Ferhat Abbas, Setif 19000, Algeria; 2 Department of Pharmaceutical Chemistry, College of Pharmacy, Jouf University, Sakaka 72341, Saudi Arabia; 3 Department of Pharmaceutics, College of Pharmacy, Umm Al-Qura University, Makkah 21955, Saudi Arabia; 4 Department of Pharmaceutics and Industrial Pharmacy, Faculty of Pharmacy, Minia University, Minia 61519, Egypt; 5 Flavour and Aroma Chemistry Department, National Research Center, Cairo 12622, Egypt

**Keywords:** MDR, *Thymus vulgaris*, *Salvia officinalis*, antibacterial activity, GC/MS, molecular docking, pus samples

## Abstract

In the context of the globally growing problem of resistance to most used antibacterial agents, essential oils offer promising solutions against multidrug-resistant (MDR) bacterial pathogens. The present study aimed to evaluate the prevalence, etiology, and antibiotic-resistance profiles of bacteria responsible for pyogenic infections in Regional Military University Hospital of Constantine. Disc diffusion and broth microdilution (MIC) methods were used to evaluate the antimicrobial activity of essential oils from five Algerian aromatic plants growing wild in the north of Algeria—*Salvia officinalis* (Sage), *Thymus vulgaris* (Thyme), *Mentha pulegium* L. (Mentha), *Rosmarinus officinalis* (Rosemary), and *Pelargonium roseum* (Geranium)—against reference and MDR strains. During three months of the prospective study, 112 isolates out of 431 pus samples were identified. *Staphylococcus aureus* was the most predominant species (25%), followed by *Klebsiella pneumoniae* (21.42%)*, Pseudomonas aeruginosa* (21%), and *Escherichia coli* (17.95%). Among pus isolates, 65 were MDR (58.03%). The radial streak-line assay showed that *R. officinalis* and *M. pulegium* L. had weak activity against the tested strains, whereas *P. roseum* showed no activity at all. Meanwhile, *T. vulgaris* was the most potent, with an inhibition zone of 12–26 mm and an MIC value ranging between 0.25 and 1.25%, followed by *S. officinalis* with an inhibition zone of 8–12 mm and an MIC value ranging between 0.62 and 2.5%. Generally, *A. baumannii* and *S. aureus* ATCC6538P were the most sensitive strains, whereas *P. aeruginosa* ATCC27853 was the most resistant strain to the oils. Gas chromatography–mass spectrometry analysis of chemical composition revealed the presence of borneol (76.42%) and thymol (17.69%) as predominant in thyme, whereas camphor (36.92%) and α- thujone (34.91%) were the major volatiles in sage. The in-silico study revealed that sesquiterpenes and thymol had the highest binding free energies against the vital enzymes involved in biosynthesis and repair of cell walls, proteins, and nucleic acids compared to monoterpenes. The results demonstrated that *T. vulgaris* and *S. officinalis* are ideal candidates for developing future potentially active remedies against MDR strains.

## 1. Introduction

There is an ever-increasing demand for plant-based therapeutics in both developing and developed countries due to a growing recognition by customers of natural products, which are readily available at affordable prices with almost no complications, and of avoiding the side-effects of chemotherapeutic agents like hypersensitivity, immune-suppression, and allergic reactions [1]. Many studies have reported essential oils’ (EOs) antimicrobial and antifungal activity against various microorganisms, including pathogenic Gram-negative and Gram-positive bacteria. For instance, the presence of long-chain (C6–C10) alcohols and aldehydes in cilantro’s EO made it efficient against *Listeria monocytogenes* [2]. At the same time, oregano and thyme EO are both effective at reducing or preventing the growth of *E. coli* O157:H7 in food [3]. Volatile constituents of EOs, like terpenes and phenolic components, e.g., thymol, carvacrol, and eugenol, are responsible for the reported antibacterial activity [4]. The Algerian flora is rich in thousands of wild herbs with medicinal properties like antimicrobial, antifungal, antioxidant, anticancer, and many other properties reported extensively in the literature based on their bioactive EOs and extracts [5,6].

The rise in infections by multidrug-resistant bacteria (MDR) in recent years has increased interest in the possible antibacterial activities of medicinal plants and their metabolites. MDR pathogens like *Pseudomonas aeruginosa* commonly cause hospital-associated infections, such as *Acinetobacter baumannii*, extended spectrum-beta-lactamase (ESBL)-producing *Escherichia coli*, and carbapenemase-producing *Klebsiella pneumoniae*, not to mention the possibility of transmission in the community [7,8]. Such bacterial isolates may be immune to every antibiotic currently on the market [9]. Creating new antimicrobials is one strategy to combat the medication-resistance issue; in this context, EOs are being researched for their antibacterial properties. To our knowledge, only *Origanum glandulosum* Desf, *Artemisia judaica* L. [6,10], and four Lamiaceae aromatic plants (*Thymus capitatus* L., *Lavandula dentata* L., *Salvia officinalis* L., and *Mentha rotundifolia* L.) [1] from the Algerian flora were screened against MDR. However, most studies dealing with the antibacterial activity of other Algerian EOs were based on non-pathogenic or reference strains [11].

This study aimed to determine the antimicrobial activity of some Algerian EOs, namely, *Rosmarinus officinalis*, *Thymus vulgaris*, *Salvia officinalis*, *Pelargonium roseum*, and *Mentha pulegium*, against MDR bacteria isolated from pus samples. The most potent EOs were analyzed using gas chromatography–mass spectrometry (GC–MS). The mechanism of EOs’ antibacterial action against MDR could be elucidated using cheminformatics [12]. Molecular docking is a cheminformatics tool often used to gain valuable insight into the possible molecular mechanisms of pharmacologically active substances. Herein, molecular docking was employed to identify a possible mechanism of action correlated with the recorded antimicrobial effect of the potent EO components against target proteins associated with bactericidal/bacteriostatic effects, such as DHPS, DHFR, Ddl, penicillin-binding protein 1a PBP1a, DNA gyrase, type-IV topoisomerase, and isoleucyl-tRNA synthetase (IARS) [13]. The search for safe botanical materials in this work presents an excellent opportunity to use EOs as antibacterial agents in the pharmaceutical industry and clinical settings to treat or prevent MDR-strain infections.

## 2. Results and Discussion

### 2.1. Prevalence of Bacterial Isolates

**Figure 1:**
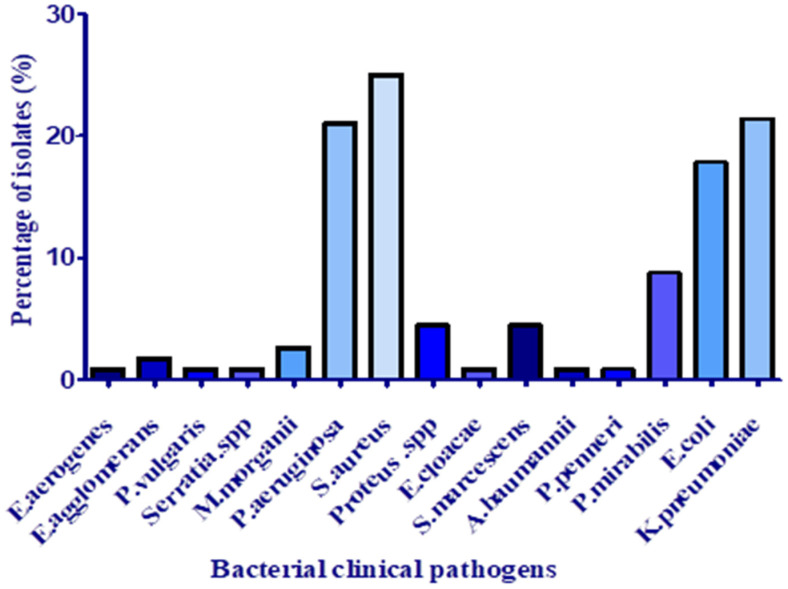
Distribution of species isolated from pus samples.

During the collection period of three months, 112 isolates out of 431 pus samples were identified, which constitute 34.67% of the total isolates detected in different samples collected from the hospital departments (pus, hemoculture, CSL, and urine). *S. aureus* was the pathogen most commonly isolated from pus specimens with a frequency of 25% (Figure 1). This finding agreed with those of Tchakal-Mesbahi et al. [14], who investigated pus from wound infections in the burn center at Mouhamed Seghir Nekkache Military Hospital of Algiers with a frequency of 28.22%. In this study, *K. pneumoniae* was the second most predominantly isolated pathogen (21.42%), followed by *P*. aeruginosa (21%) and *E. coli* (17.95%). According to Hak-Jae et al. [15], *Proteus spp*., *S. aureus*, and *K. pneumoniae* are the most common bacteria from the pus of wound infections. These bacterial species are abundant in the skin and easily enter the surgical site during hospitalization. At the same time, other species were isolated with less frequency, as presented in Figure 1: *Proteus mirabilis* (8.92%), *Proteus spp*., and *S. marcescens* (4.46%).

### 2.2. Distribution of Bacterial Isolates According to the Hospital Department

**Figure 2:**
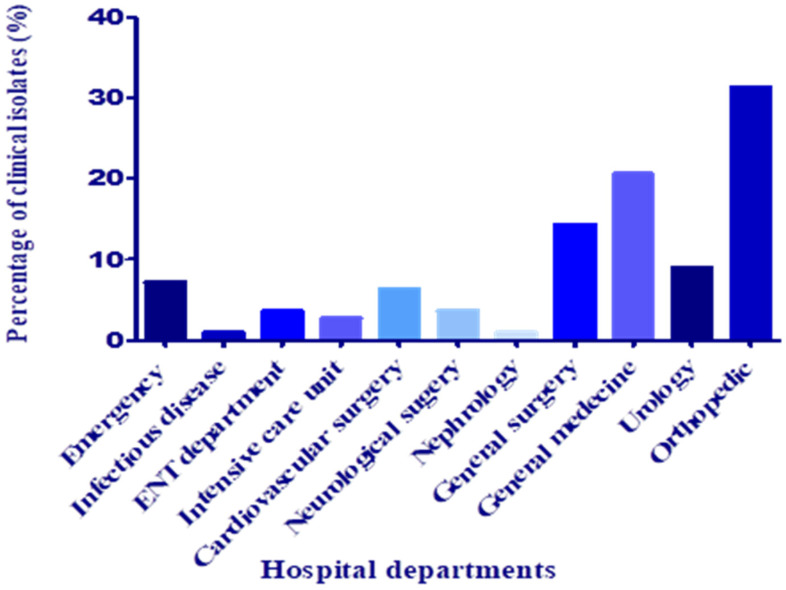
Distribution of clinical isolates according to the hospital department.

Pus samples were recuperated mainly from wound, post-surgical, ear, and ODP (obstructive pulmonary disease) infections. Most of the samples were collected from the orthopedic department and general medicine, followed by general surgery, with frequencies of 31.25, 20.53, and 14.28%, respectively (Figure 2). According to Uçkay et al. [16], in the orthopedic department, surgical site and osteoarticular infections, in addition to bone trauma, are frequent and challenging to treat and associated with lifelong recurrence risks of around 10–20%.

### 2.3. Distribution of Bacterial Isolates According to Sex

The distribution of the total isolates (112) according to sex showed a vast difference between the frequency of isolation from male samples (84.82%) and that derived from female samples (15.17%), with a sex ratio of 5.58 male/female. These results are consistent with those obtained by Lye et al. [17], whose study showed a predominance of isolates derived from males (57%) hospitalized for a longer duration before bacteremia or lodged in the ICU and who were more acutely ill.

### 2.4. Antibiotics-Susceptibility Testing

The testing of methicillin-resistant *S. aureus* (MRSA) susceptibility to most used antibiotics showed a very high resistance frequency to penicillin G (50%), oxacillin, and cefoxitin (46.4%), whereas they were less resistant to erythromycin and spiramycin (10.71%) (Table 1). Lincomycin, clindamycin, amikacin, and ofloxacin were the most effective antibiotics. The present study’s resistance frequencies were lower than those obtained by Feleke et al. [18] at Gondar Specialized Hospital in Ethiopia, where *S. aureus* isolates (35.6%) were the most common isolates, with an MRSA-isolation rate of 67.5%. The most common clinical samples were wounds/pus (63%), which agrees with the present study. They reported very high resistance rates to penicillin (96.1%), amoxicillin (50.6%), and clindamycin (36.4%) [18]. The variations in resistance-frequency rates may be due to host, microbial, and environmental factors. This major human pathogen mostly comes from the patient themself, but it is mainly transported to infectious sites by personnel or medical devices. Therefore, researchers continue to seek to understand these infections’ dynamics and their resistance mechanisms to prevent and cure them [19]. In the current study, Enterobacteriaceae members were mainly resistant to ampicillin, amoxicillin, amoxicillin/clavulanate, and third-generation cephalosporins (cefotaxime and ceftriaxone), whereas imipenem and amikacin were active on all isolates (0%). Many studies reported a high resistance level against penicillins, cephalosporins, and trimethoprim/sulfamethoxazole [20]. Whereas *P. aeruginosa* demonstrated increased susceptibility to the most used antibiotics, including ticarcillin/clavulanate, piperacillin, and ceftazidime (8.33%), all isolates were susceptible to colistin (0%). In contrast, isolates of *A. baumannii* were the most resistant. They were fully resistant to all tested antibiotics (100%) except for amikacin and colistin (0%). These findings align with those of Tchakal-Mesbahi et al. [14], who reported that A. baumannii isolates were highly resistant to ceftazidime and imipenem (90%), and more than 80% were resistant to amikacin, gentamicin, and ciprofloxacin. According to Lin and Lan [21], *A. baumannii* is known for its intrinsic resistance to antibiotics and ability to acquire gene-encoding resistance determinants.

### 2.5. Phenotypic Detection of Multidrug-Resistant Bacteria

The analysis of susceptibility results by antibiogram showed that 65 out of 112 tested isolates (58.03%) were resistant to at least three antibiotics from different classes (MDR phenotype), which indicates an alarming frequency. Dessie et al. [22] reported similar results with a high frequency of MDR (75.2%) in a study conducted in Addis Ababa. The increase in the MDR phenotype greatly impacts public health by increasing treatment failure, which requires approaches to be developed for rational use of antimicrobials; improving hand hygiene and infection control; and developing new alternatives for commonly used antibiotics. The difference in the prevalence of MDR is associated with many factors, including long-term antimicrobial therapy, cross-transmission, length of hospital stays, invasive procedures, differences in environmental conditions, and policy of antibiotics use [23,24]. Antimicrobial susceptibility patterns often differ between geographical regions, populations, and hospital types/units [25,26].

Resistance to third-generation cephalosporins is mainly due to ESBL production; in this study, 22.61% were Enterobacteriaceae-producing ESBL. This frequency was less than that reported by Elmekes et al. [27]. They showed that 48% of Enterobacteriaceae were ESBL producers in a study conducted at the intensive care unit of the University Hospital Center in Marrakesh, Morocco. Resistance to cephalosporins also may be due to AmpC cephalosporinase production; in this study, only 2.38% of Enterobacteriaceae-producing cephalosporinases were identified. The incidence of MRSA was also alarming, with a frequency of 46.42 %. Similar results were obtained by Nigussie et al. and Latif et al. [28,29], of 38.5% and 31.25%, respectively. These variations may be due to the type of assessment used for the diagnosis of MRSA and the status of the hospital [30]. Therefore, it is essential to confront the rapid increase in infections caused by MRSA and Enterobacteriaceae-producing ESBLs in Algeria.

### 2.6. Antibacterial Activity

#### 2.6.1. The Radial Streak-Line Method

Analysis of radial streak-line assay results showed that, among five tested essential oils, *T. vulgaris* oil was the most active, followed by *S. officinalis* (Table 2). However, *P. roseum*, *M. pulegium*, and *R. officinalis* showed weak activity. These findings led to the selection of *T. vulgaris* and *S. officinalis* essential oils for further investigation; disk diffusion assay, GC/MS, and in silico study revealed the volatile constituents responsible for the activity against enzymes involved in biosynthesis and repair of cell walls, proteins, and nucleic acids of the MDR pathogens.

#### 2.6.2. Evaluation of the Antibacterial Activity of Potent EOs

The antibacterial activity of *T. vulgaris* and *S. officinalis* essential oils (EOs) were evaluated by the disc-diffusion method, minimum inhibitory concentration (MIC), and minimal bactericidal concentration (MBC), as shown in Table 3. The results show that *T. vulgaris* EO was very effective against *S. aureus* ATCC 6538P and *A. baumannii* with inhibition-zone diameters of 26 mm and 22 mm, respectively. It was also active on MRSA, *S. marcescens* (17 mm), and *E. coli* ESBL (15 mm). The previous results indicate that *T. vulgaris* EO had a broad spectrum against Gram-negative and Gram-positive bacteria, except for P. aeruginosa. MIC values ranged from 0.07 to 1.25% and for MBC values between 0.07 and 2.5%. The highest antibacterial activity of *T. vulgaris* EO is mainly due to the presence of phenolic compounds and monoterpene hydrocarbons [31].

The antibacterial activity of *S. officinalis* essential oil showed a moderate effect against clinical isolates and *S. aureus* ATCC 6538P with an inhibition zone ranging from 8 to 12 mm. The results obtained are different from those reported by Moumni et al. [32], who found a more potent antimicrobial activity against *E. coli* (11.3 mm), *B. subtilis* (17.3 mm), and *P. aeruginosa* (7 mm). This variation may be attributed to many factors, including species, plant origin, climatic conditions, distillation method and conditions, chemical composition, time of harvesting and storage conditions, and bacterial-strain susceptibility [33,34,35]. MIC values ranged between 0.62 and 2.5%, whereas MBC values varied from 2.5 to 5%. In previous studies, Delamare et al. [36] attributed the antibacterial activity of S. officinalis EO against *E. coli, P. aeruginosa, B. subtilis,* and *S. aureus* to high concentrations of thujone, 1,8-cineole, and camphor.

### 2.7. Chemical Composition of the Potent EOs

**Figure 3:**
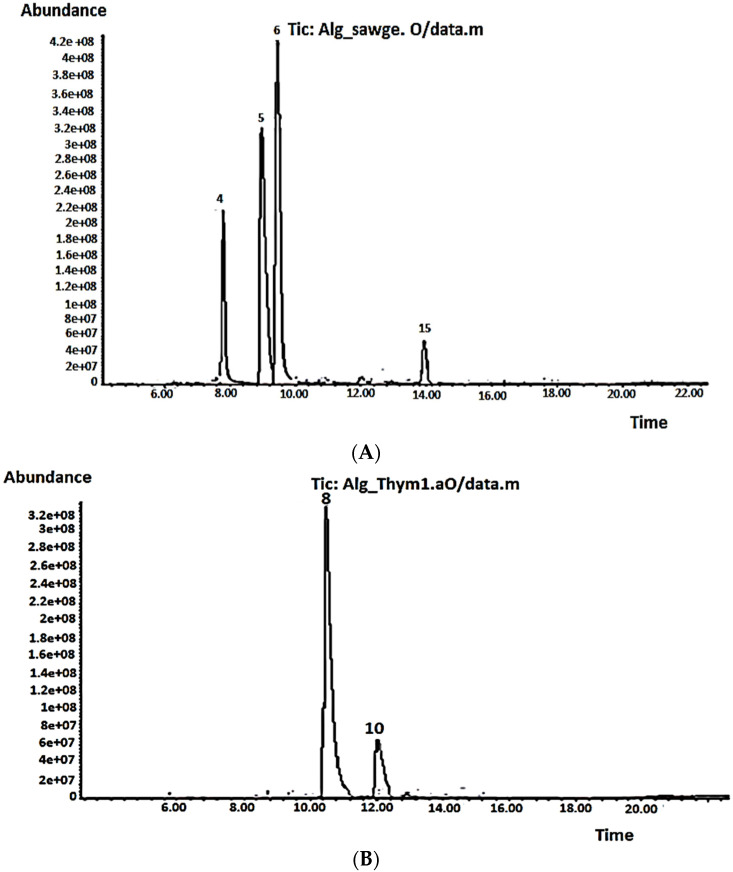
Volatile chromatograms for (**A**) *S. officinalis* and (**B**) *T. vulgaris*.

The chemical composition of volatile oils from aerial parts of *S. officinalis* and *T. vulgaris* was characterized by GC-MS (Table 4, Figure 3A,B). A total of 14 and nine components were identified, representing 99.56 and 97.83% of the total oils, respectively. Camphor (39.62%), α-thujone (34.91%), 1,8-cineole (13.20%), and viridiflorol (5.84%) were the dominant compounds in the S. officinalis oil (Table 4, Figure 3A). The previous findings are consistent with Dob et al. [37], Boutebouhart et al. [38], and Mahdjoubi et al. [39], who identified the same trend in oils extracted from plants cultivated in the Algiers and Laghouat regions, Algeria, despite remarkable quantitative differences due to geographical variation. The chemotaxonomy of familiar sage *(Salvia officinalis)* based on the volatile constituents studied by Craft et al. [40] revealed five major chemotypes, with the most common being an α-thujone/camphor/1,8-cineole, α-humulene, β-thujone, 1,8-cineole/camphor, and sclareol/α-thujone. The *S. officinalis* under investigation and most of the Algerian oils reported in the literature belonged to the typical α-thujone/camphor/1,8-cineole chemotype.

In the volatile oil extracted from *T. vulgaris*, nine compounds were identified, representing 97.83% of the total oil, with the major constituents being borneol (76.42%) and thymol (17.69%) (Table 4, Figure 3B). According to Benaliouche et al. [41], the genus Thymus contains more than 300 species, 11 of which are located in Algeria. Based on the chemotype, each species showed variability in the chemical profile of their EOs, which affected their physicochemical and biological properties. Several chemotypes have been characterized based on the terpenes, including linalool, borneol, geraniol, sabinene hydrate, thymol, and carvacrol [42]. Consequently, to our knowledge, the Algerian thyme oil under investigation in the current study represents the first reported one to belong to the borneol chemotype. Quantitative and qualitative variations in the *T. vulgaris* EOs obtained from different regions in Algeria have been reported in the literature based on their chemotype. For example, Benaliouche et al. [41] identified thyme oil extracted from Annaba as linalool chemotype, whereas Abdelli et al. [43] showed that EOs extracted from *T. vulgaris* cultivated in Tlemcen and Mostaganem belong to the carvacrol chemotype. Interestingly, in a previous study by Abdelli et al. [44], thyme EOs from the same regions, Tlemcen and Mostaganem, had thymol as the predominant EO, at 59.5 and 67.3%, respectively. Generally, the variations in the chemical composition of thyme EO may be attributed to many factors, such as climatic change, environmental conditions, plant age, plant part, development stage, growing place, harvesting period, or principal chemotype, as they all affect plant biosynthetic pathways and consequently the relative proportion of the main ingredients [45].

### 2.8. Molecular-Docking Study

In silico assessment employing a molecular-docking approach was used to better understand the molecular basis of the antibacterial activity provided by the major volatile components of the potent EOs (*S. officinalis* and *T. vulgaris*), as antibiotics target the microbial metabolism and restrict their growth by deactivating the vital enzymes involved in biosynthesis and repair of cell walls, proteins, and nucleic acids such as isoleucyl-tRNA synthetase, DNA gyrase, dihydropteroate synthase, D-alanine ligase, topoisomerase 4, dihydrofolate reductase, and penicillin-binding protein (Table 5).

Aminoacyl-tRNA synthetases have been identified as possible drug targets for several infectious diseases. They are responsible for charging a specific tRNA with its cognate amino acid, which is essential for protein synthesis. Inhibition of tRNA aminoacylation has proven to be an effective antimicrobial strategy, impeding a necessary step of protein synthesis. The design of novel aminoacylation inhibitors is complicated by either the presence of MDR clinical isolates or the steadfast requirement to avoid off-target inhibition of protein synthesis in human cells [46]. The major sesquiterpenes in both potent EOs are β- caryophyllene (−6.8 kcal/mol), α-humulene (−7.0 kcal/mol), and viridoflorol (−7.0 kcal/mol), and revealed the highest binding affinity against isoleucyl-tRNA synthetase (PDB ID: 1JZQ) compared to monoterpenes (Table 5). The previous findings agree with Jianu et al. [13], where caryophyllene oxide showed the highest affinity toward 1JZQ during in silico and in vitro evaluation of the antimicrobial and antioxidant potential of Romanian *Mentha × smithiana* R. Graham EO.

DNA gyrase controls the topology of DNA during transcription and replication by introducing transient breaks to both DNA strands. Since DNA gyrase is pivotal for bacterial survival, it is essential to exploit bacterial DNA gyrase as a critical target for the antibacterial agents. Consequently, a molecular-docking study was carried out to examine the binding interactions of the major volatile constituents with the pocket of DNA gyrase (PDB ID: 1KZN) (Table 5). The compounds displayed binding energy ranging from −4.5 to −6.3 kcal/mol for thymol, one of the major constituents in *T. vulgaris* EO (Table 5). The extract components of *Ocimum cufodontii* used traditionally in Ethiopia as an antibacterial agent shown a comparable binding affinity toward DNA gyrase (−6.1 to −6.9 kcal/mol), which is in line with the results of the present study [47]. The inhibition of dihydrofolic acid-formation affects DNA synthesis. Therefore, antibiotic agents compete with p-amino benzoic acid to bind with the dihydropteroate synthetase (PDB ID: 2VEG) with much greater affinity and inhibit the formation of dihydrofolic acid. In line with the affinity showed by β-cubebene towards 2VEG (−6.1 kcal/mol) reported by Salvi et al. [48], the sesquiterpenes in the current study—α-humulene and viridoflorol—showed similar affinity (Table 5).

Regarding the target-protein structures involved in antimicrobial activity, the results indicate an increased affinity of thymol (−7.7 kcal/mol) towards the D-alanine–D-alanine ligase (PDB ID: 2ZDQ). The enzyme 2ZDQ catalyzes the condensation of two D-Ala molecules using ATP to produce D-Ala–D-Ala, the terminal peptide of a peptidoglycan monomer. The cell-wall peptidoglycan polymer is produced through cross-linking peptidoglycan monomer units [49]. The highest affinity of thymol among the examined volatiles (Table 5), which seems to have been responsible for the higher antibacterial activity of *T. vulgaris* EO, is due to its bactericidal effect by bacterial cell-wall denaturation, causing leakage of essential nutrients [50]. Therefore, the assumption that one of the antibacterial mechanisms of the EO’s monoterpene components is the suppression of bacterial-wall synthesis by inhibiting the D-alanine–d-alanine ligase enzyme is highly plausible. The binding-affinity energy of thymol recorded in the current study was superior compared to the literature [13,48] and opens the prospects for using EOs rich in thymol as antibacterial agents against MDR isolates. In Gram-positive bacteria, topoisomerase 4 catalyzes the separation of daughter strands following replication. The molecular-docking analysis for the major volatiles in the potent antibacterial EOs of the present study revealed that 1,8-cineole and sesquiterpenes showed the highest binding affinity towards topoisomerase 4 (PDB ID: 3RAE), with −6.0 to −6.5 kcal/mol (Table 5), which is comparable to the binding affinity of *O. gratissimum*, *O. tenuiflorum*, and *O. sanctum* EOs volatiles reported by Salvi et al. [48] and higher than the volatiles of *Mentha × smithiana* essential oil [13].

Dihydrofolate reductase (PDB ID: 3SRW) is an enzyme in the thymidine synthesis pathway. Antibiotics bind to 3SRW and inhibit thymidine synthesis, which eventually affects DNA synthesis. In agreement with Jianu et al. [49] and Salvi et al. [48], sesquiterpenes exhibited higher in silico binding affinity towards 3SRW than well-known antibiotics used against enzymes such as trimethoprim. Viridiflorol had the highest binding energy (−8.1 kcal/mol) among all the examined volatiles towards all receptors included in the study. An essential part of the bacterial cell wall, cross-linked peptidoglycan, is created by penicillin-binding proteins (PDB ID: 3UDI). These proteins feature penicillin-sensitive C-terminal transpeptidase and N-terminal trans-glycosylase domains. Therefore, the inactivation of 3UDI proteins limits the development of cell walls, which eventually causes bacterial growth to cease [51]. In silico analysis revealed that the three phytoconstituents—viridoflorol (6.5 kcal/mol), α-humulene (6.0 kcal/mol), and β-caryophyllene (5.9 kcal/mol)—had a comparatively similar binding affinity towards 3UDI to that of β-cubebene, caryophyllene oxide, and ylangene, as reported by Salvi et al. [48].

**Figure 4:**
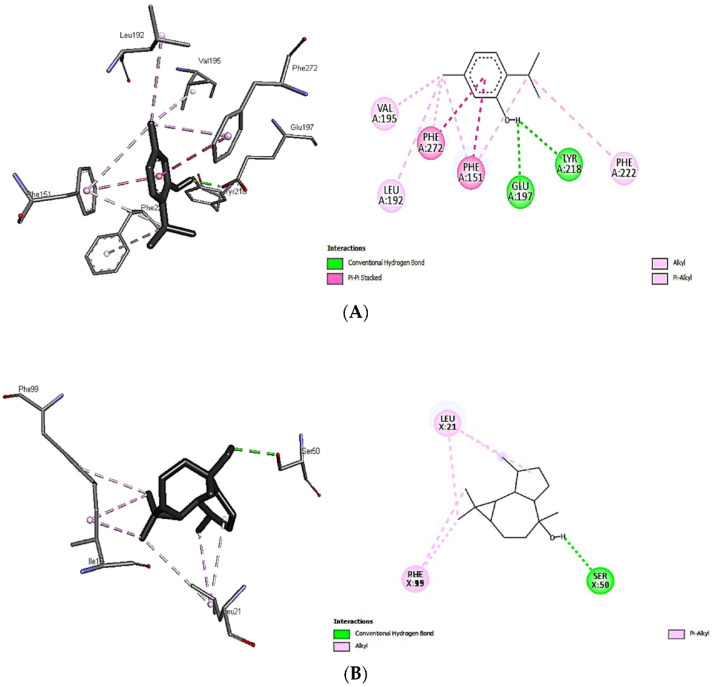
Interactions of thymol with D-alanine: D-alanine ligase 2ZDQ (**A**) and viridoflorol with dihydrofolate reductase 3SRW (**B**).

Since thymol (−7.7 kcal/mol) and viridoflorol (−8.1 kcal/mol) were recorded, the best docking scores among all other volatiles examined were towards 2ZDQ and 3SRW; therefore, binding analysis was performed to reveal the interaction between ligands and protein-binding sites (Figure 4A,B). The hydroxyl group of thymol was very well oriented, forming two conventional hydrogen bonds with TYR A:218 and GLU A:197 of 2ZDQ. In addition, thymol showed other pi-alkyl interactions with PHE A:151, 222, 272; pi-pi stacked with PHE A:151, 272; and finally, alkyl binding with protein moieties like VAL A:195, LEU A:192, and others (Figure 4A). On the other hand, the interaction of viridoflorol with 3SRW involved the amino acids residues VAL7, ALA8, ILE15, GLY16, PHE17, ASN19, GLN20, LEU21, LYS46, THR47, ILE51, PHE93, GLY94, GLY95, GLN96, PHE99, and THR122 through alkyl and pi-alkyl interactions. A conventional H-bonding with SER50 was detected through in-silico analysis (Figure 4B). The previous findings revealed the responsibility of different binding interactions for the higher docking scores of both thymol and viridoflorol, and consequently the antibacterial activity of both *S. officinalis* and *T. vulgaris* against MDR clinical isolates.

## 3. Materials and Methods

### 3.1. Study Design

During three months of a prospective study conducted at the central microbiology laboratory in the Regional Military University Hospital of Constantine, a total of 112 isolates from 431 pus samples of hospitalized patients was obtained and analyzed.

### 3.2. Identification of Clinical Isolates

Identification of isolated bacteria from pus samples was conducted according to their morphological and biochemical characteristics. Pus smeared on a clean, grease-free slide showing Gram-positive staining and occurring as clusters was subjected to growth on mannitol salt agar, coagulase, and catalase tests [52,53], whereas that showing Gram-negative stains was purified on differential and selective media. The species were identified using a qualitative micro-method employing conventional and chromogenic substrates to identify clinically important selected oxidase-negative. Identification kit RapID™ ONE system tests and Remel™ RapID™ ERIC™ software v.1.0.771 (Thermo Scientific, Waltham, MA, USA) were also used to identify isolates according to the recorded database.

### 3.3. Antibiotic-Susceptibility Test

Susceptibility to antibiotics was carried out using the disk-diffusion method on Muller Hinton Agar at 37 °C for 18 h. Results were interpreted according to CLSI breakpoint criteria (2014). Three reference isolates (*Staphylococcus aureus* ATCC 25923, *Escherichia coli* ATCC 25922, and *Pseudomonas aeruginosa* ATCC 27853) were used for quality control of the purchased disks (Oxoid, Basingstoke, Hampshire, UK). *S. aureus* isolates were tested on a total of 10 different antibiotic disks: oxacillin (OX5), cefoxitin (FOX30), penicillin G (P10), spiramycin (SP100), clindamycin (DA2), lincomycin (L15), erythromycin (E15 (RD5), vancomycin (VA30), amikacin (AK30), and ofloxacin (OFX5), whereas *Enterobacteriaceae* members were tested for amoxicillin (AMX25), amoxicillin/clavulanate (AMC10), cefotaxime (CTX30), ceftriaxone (CRO 30), ampicillin (AMP10), cefazolin (CF30), imipenem (IMP10), ofloxacin (OFX5), amikacin (AK10), and trimethoprim/sulfamethoxazole (SXT1.25/23.75). Non-fermentative bacilli (*P. aeruginosa and A. baumannii*) were tested for the following antibiotics: piperacillin (PIP100), ticarcillin (TIC75), ticarcillin–clavulanate (TCC75), aztreonam (ATM30), ceftazidime (CAZ30), piperacillin (PRL100), levofloxacin (LEV5), amikacin (AK30), colistin (CT10), and trimethoprim/sulfamethoxazole (SXT1.25/23.75).

### 3.4. Phenotypic Detection of MDR-Strain Resistance Mechanisms

Resistance mechanisms were detected phenotypically for MDR isolates based on the analysis of susceptibility to antibiotics by antibiogram and the use of inhibitors (clavulanic acid and cloxacillin). *Enterobacteriaceae* and non-fermentative gram-negative bacilli were selected based on their resistance to third-generation cephalosporins (CTX and CAZ) to detect extended-spectrum beta-lactamase (ESBL) and cephalosporinase production. A double-disk synergy test (DDST) was used to detect ESBL production; increasing inhibition-zone diameter (synergy) indicates a positive test. Positive results were confirmed by the double-disk-diffusion test (DDDT) [54], whereas AmpC cephalosporinase detection was performed for isolates with a negative synergy test using cloxacillin as an inhibitor (200 μg/mL) and cefoxitin (FOX30), as described by Tan et al. [55]. Disks of ox (5 µg) and fox (30 µg) were used for screening methicillin-resistant *S. aureus* (MRSA) [56].

### 3.5. Plant Material and Extraction

Five aromatic plants growing wild in different regions of North Algeria were collected during the flowering season (June 2019). *Thymus vulgaris* was collected from Setif, *Mentha pulegium* and *Pelargonium roseum* were collected from Constantine, and *Salvia officinalis* and *Rosmarinus officinalis* were obtained from the Bejaia region. Identification was performed by an ecologist at the biology laboratories at the Faculty of Sciences of Bejaia University. The plant’s aerial parts (leaves, flowers, and stems) were dried in the dark at room temperature (25–26 °C). The plant material (100 g) was supported above boiling water on a perforated grid for 3 hours. The condensate was recuperated from the condenser, and a glass decanter separated essential oils from the condensate. The purified oils were stored in brown glass bottles at 4 °C until their use.

### 3.6. Bacterial Strains

Four reference isolates *(P. aeruginosa* ATCC2525, *S. aureus* ATCC6538P, *K. pneumoniae* ATCC700603, *E. coli* ATCC8739) and a selection of five MDR clinical isolates from the military hospital (MRSA, *E. coli* ESBL, *K. pneumoniae* ESBL, *S. marcescens* ESBL, and *A. baumannii*) were used to evaluate the effectiveness of the extracted essential oils.

### 3.7. Antimicrobial Activity

#### 3.7.1. The Radial Streak-Line Method

This is a preliminary qualitative test generally used to check and select between efficient pure essential oils. Sterile filter discs (Whatman n°1, 6 mm diameter) were impregnated with 10 µL of stock solutions of essential oils and placed at the center of the Muller Hinton agar plate surface. Plates were inoculated with bacterial suspensions adjusted to 0.5 McF (≈1.5 × 10^8^ UFC/mL) by radial lines of inoculums from the border to the center of the plate. The antimicrobial activity was evaluated by analyzing the size in mm of the inhibition zone [57].

#### 3.7.2. Agar Diffusion Method

The antimicrobial activity of two-fold serial diluted essential oils (in dimethyl sulfoxide) was determined as previously described [17]. Briefly, a bacterial suspension (10^6^ CFU/mL) was inoculated on MH agar, then 20 μL of each oil dilution were poured into wells of 6 mm diameter, and DMSO was used as a negative control. To allow oil diffusion onto agar plates, they were incubated at 4 °C for two hours, then incubated at 37 °C overnight (24 h); the mean of inhibition-zone diameters was determined for each dilution. Each assay was repeated three times [58].

#### 3.7.3. Broth Microdilution Assay

A microdilution test was performed using 96-well microplates. The serial dilutions of the essential oils dissolved in DMSO were prepared in BHI broth with a concentration ranging from 0.04 to 5% (*v/v*). Each well containing 50 µL of diluted essential oil was inoculated with 50 µL of bacterial suspension in BHI Broth (10^6^ CFU/mL). A well containing 100 μL of BHI broth was used as a negative control and a well containing 100 μL of BHI broth with a bacterial suspension without essential oil as a positive control. After that, incubation was carried out at 37 °C for 24 h. Tetrazolium chloride (2,3,5-triphenyl-2H-tetrazolium chloride TTC) was used as an indicator of viability to determine values of minimum inhibitory concentrations (MICs). A volume of 10 μL of broth from each well with no visible growth (≥MICs) was subcultured onto fresh MH agar plates, then incubated at 37 °C for 24 h. Concentration in which there is no colony growth is considered the minimum bactericidal concentration (MBC) [59].

### 3.8. Chemical Composition of Potent EOs

Potent steam-distilled oils (SD) were analyzed using gas chromatography–mass spectrometry (GC-MS); separation was carried out on a Trace GC Ultra Chromatography system (Thermo Scientific, Waltham, MA, USA) outfitted with an ISQ mass spectrometer and a 60 m 0.25 mm 0.25 μm TG-5MS capillary column (Thermo Scientific, Waltham, MA, USA). The column-separation-program temperature began at 50 °C with a holding time of 3 min, then was raised by 4 °C per minute to 140 °C with a holding period of 5 min. After that, the temperature rose at a rate of 6 °C per minute before reaching 260 °C for an isothermal holding period of 5 min. The injector temperature was 180 °C, the ion source temperature was 200 °C, and the transition-line temperature was 250 °C. Helium flowed at a steady rate of 1.0 mL/min as the carrier gas. The mass spectrometer had an ionization energy of 70 eV and a scan range of 40–450 *m/z*. The MS computer library (NIST library, 2005 edition), raw chemicals, and published data were used to identify compounds [40,60]. Integration by GC was used to compute the area percentage of the detected components. By comparing the values with those reported in the literature and utilizing the retention durations of a homologous series of C_6_–C_26_ n-alkanes, the Kovats index was determined for each compound [61].

### 3.9. Molecular Docking

The crystal structures of isoleucyl-tRNA synthetase (PDB ID: 1JZQ), DNA gyrase (PDB ID: 1KZN), dihydropteroate synthase (PDB ID: 2VEG), D-alanine: D-alanine ligase (PDB ID: 2ZDQ), IV topoisomerase (PDB ID: 3RAE), dihydrofolate reductase (PDB ID: 3SRW), and penicillin-binding protein 1a (PDB ID: 3UDI) were obtained from the Protein DataBank (https://www.rcsb.org/, accessed on 16 August 2022). It was prepared as a receptor by removing water and co-crystallized ligands and ions, then protonated using the Pymol software ver. 2.5.1 (Supplementary files as receptors in pdb format). Meanwhile, the 3D structures of the ligands, which were downloaded from the PubChem database (http://pubchem.ncbi.nlm.nih.gov/, accessed on 16 August 2022), were optimized by using the MMFF94 force field by Avogadro Software ver. 1.2.0 (Supplementary files as ligands in sdf and mol2 formats). Blind docking was performed using a web-based program called CB-DOCK2 (http://clab.labshare.cn/cb-dock/php/, accessed on 16 August 2022). After submission, CB-Dock2 checks the input files and converts them to pdbqt-formatted files using OpenBabel and MGL Tools. Next, CB-Dock predicts cavities of the protein and calculates the centers and sizes of the top N (n = 5 by default) cavities. Each center and size and the pdbqt files are submitted to AutoDock Vina for docking. The final results are displayed after the computation of N rounds (Supplementary docking files in pdb format). The benchmarks performed by Liu et al. [62] illustrated that, in terms of success rates for top-ranking poses whose root-mean-squared deviation (RMSD) was within 2 Å from the position in the X-ray crystal structure, CB-Dock2 outperformed other blind-docking tools. The profiles of interaction and visualization were performed for the best-docked complexes using Discovery Studio software (Ver. 21.1.0.20298) [63].

## 4. Conclusions

The preliminary antibacterial examination of many Algerian essential oils growing wild in the northern regions against MDR bacterial pathogens revealed the efficacy of *S. officinalis* and *T. vulgaris*, which was assured in vitro using further advanced assays. The higher antibacterial activities of the previous essential oils aligned with their higher concentrations of camphor, α-thujone, viridoflorol, borneol, and thymol. The in-silico study showed the higher affinity of sesquiterpenes and thymol toward vital enzymes involved in the biosynthesis and repair of cell walls, proteins, and nucleic acids of the MDR bacteria of *S. officinalis* and *T. vulgaris* compared to monoterpenes. The outcomes showed that *T. vulgaris* and *S. officinalis* are excellent prospects for creating potential future treatments that are active against MDR strains as alternatives for the standard used antibiotics.

## Figures and Tables

**Table 1:** Antimicrobial-susceptibility profile of multidrug-resistant bacteria (MDR) against most commonly used antibiotics.

Isolates	Antibiotics (%)									
MRSA	P	OX	FOX	E	SP	L	DA	OFX	VA	AK
	50	46.42	46.4	10.71	10.71	1.78	1.78	2.67	0	3.57
	AMP	AMX	AMC	CF	CTX	CRO	IMP	AK	OFX	STX
*M. morganii*	100	100	100	100	33.3	33.3	0	0	66.6	66.6
*P. mirabilis*	20	20	10	10	10	10	0	0	20	10
*P. penneri*	100	100	100	100	0	0	0	0	100	100
*P. vulgaris*	100	100	0	100	0	0	0	0	0	100
*Serratia* spp.	100	100	100	100	0	0	0	0	0	0
*E. aerogene*	100	100	100	100	100	100	0	0	0	100
*S. marcescens*	100	100	100	100	40	40	0	0	20	40
*Proteus* spp.	80	80	80	80	0	0	0	0	40	60
*E. coli*	50	45	45	30	25	25	0	0	35	45
*K. pneumoniae*	58.3	58.3	50	58.3	50	50	0	0	41.66	54.16
	TIC	TCC	PRL	CAZ	AZM	IMP	AK	LEV	CT	STX
*P. aeruginosa*	12.5	8.33	8.33	8.33	8.33	4.16	4.16	8.33	0	12.5
*A. baumannii*	100	100	100	100	100	100	0	100	0	100

MRSA: methicillin-resistant *S. aureus*, ATBs: antibiotics, P: penicillin, OX: oxacillin, FOX: cefoxitin, E: erythromycin, SP: spriramycin, L: lincomycin, DA: clindamycin, OFX: ofloxacin, VA: vancomycin, AK: amikacin, AMP: ampicillin, AMX: amoxicillin, AMC: amoxicillin/clavulanate, CF: cefazolin, CTX: cefotaxime, CRO: ceftriaxone, IMP: imipenem, STX: trimethoprim/sulfamethoxazole, TIC: ticarcillin, TCC: ticarcillin-clavulanate, PRL: piperacillin, CAZ: ceftazidime, AZM: aztreonam, LEV: levofloxacin, CT: colistin.

**Table 2:** Effect of tested essential oils on radial bacterial growth.

Strains	Bacterial-Growth Inhibition by Essential Oils				
	*R. officinalis*	*T. vulgaris*	*S. officinalis*	*P. roseum*	*M. pulegium*
*E. coli* ATCC25953	+	+	-	-	-
*K. pneumoniae* ATCC700603	+	-	-	-	-
*S. aureus* ATCC6538P	+	+++	++	-	+
*P. aeruginosa* ATCC 27255	-	-	-	-	-
*E. coli* 2793ESBL	-	++	+	-	-
MRSA 1392	-	++	+	-	+
*A. baumannii* 2873	-	+++	-	-	+
*S. marcescens* 1393ESBL	-	+++	+	-	+
*K. pneumoniae* 3466ESBL	-	++	-	-	-

-: No inhibition; + ≤ 2 cm; ++ 2 < d ≤ 3; +++ 3 < d ≤ 4; d: inhibition zone.

**Table 3:** Antibacterial activity of *T. vulgaris* and *S. officinalis* EOs against multidrug-resistant isolates using three methods (disk-diffusion method, MIC, and MBC).

Strains	*T. vulgaris*			*S. officinalis*		
	IZ * (mm)	MIC (%)	MBC (%)	IZ (mm)	MIC (%)	MBC (%)
*E. coli ESBL*	15	0.15%	0.15%	8	2.5%	5%
*K. pneumoniae ESBL*	12	0.07%	0.07%	8	2.5%	5%
*S. marcescens ESBL*	17	0.62%	1.25%	8	2.5%	2.5%
*A. baumannii*	22	0.31%	0.62%	12	0.62%	>5%
MRSA	17	0.15%	0.15%	10	2.5%	>5%
*P. aeruginosa* ATCC27853	NA **	/ ***	/	NA	/	/
*E. coli* ATCC25953ESBL	14	1.25%	2.5%	NA	/	/
*K. pneumoniae* ATCC700603ESBL	12	1.25%	2.5%	NA	/	/
*S. aureus* ATCC6538P	26	0.62%	2.5%	12	1.25%	5%

* IZ: inhibition zone, ** NA: not active, *** /: not tested.

**Table 4:** Volatile constituents identified from Algerian *S. officinalis* and *T. vulgaris* using GC-MS.

S/N	Compound	RI ^a^	LRI ^b^	*Salvia officinalis* ^c^	*Thymus vulgaris*	Identification Method ^d,e^
1	α-Pinene	934	939	0.18 ± 0.02	0.14 ± 0.01	KI, MS
2	Camphene	951	954	0.52 ± 0.04	0.53 ± 0.03	KI, MS
3	β-Pinene	981	979	0.22 ± 0.01	0.51 ± 0.04	KI, MS
4	1,8-Cineole	1033	1031	13.20 ± 0.7	-	KI, MS, STD
5	α-Thujone	1107	1102	34.91 ± 1.7	-	KI, MS, STD
6	Camphor	1143	1146	39.62 ± 1.1	0.21 ± 0.01	KI, MS, STD
7	Isoborneol	1157	1160	0.32 ± 0.01	-	KI, MS
8	Borneol	1162	1169	0.87 ± 0.03	76.42 ± 1.13	KI, MS, STD
9	Thymol, methyl ether	1234	1235	-	0.38 ± 0.05	KI, MS
10	Thymol	1291	1290	-	17.69 ± 0.61	KI, MS, STD
11	β-Caryophyllene	1416	1419	1.09 ± 0.06	1.09 ± 0.05	KI, MS, STD
12	α-Humulene	1457	1454	1.40 ± 0.05	-	KI, MS
13	δ-Cadinene	1524	1523	0.50 ± 0.02	-	KI, MS
14	Caryophyllene oxide	1585	1583	0.17 ± 0.01	0.86 ± 0.03	KI, MS
15	Viridoflorol	1593	1592	5.84 ± 0.15	-	KI, MS, STD
16	Humulene oxide	1602	1600	0.72 ± 0.03	-	KI, MS
Total				99.56%	97.83%	-

^a^ RI: retention indices calculated on the DB-5 column using alkane standards; ^b^ LRI: retention indices according to the literature; ^c^ values represent averages ± standard deviations for triplicate experiments; ^d^ confirmed by comparison with the mass spectrum of the authentic compound; ^e^ identification by comparison with data obtained from the NIST mass-spectra library.

**Table 5:** Binding free-energy values calculated through the molecular docking of the major volatile constituents of S. officinalis and *T. vulgaris* EOs and the bacterial key metabolic enzymes as receptors.

Ligand	Binding Free Energy ΔG (kcal/mol)						
	1JZQ *	1KZN	2VEG	2ZDQ	3RAE	3SRW	3UDI
1,8-Cineole **	−5.6	−4.5	−4.5	−6.3	−6.1	−5.8	−4.9
** **α-Thujone** **	−5.8	−5.2	−4.6	−6.6	−5.9	−5.7	−4.9
** **Camphor** **	−5.4	−4.5	−4.5	−5.9	−5.4	−6.1	−5.3
** **Borneol** **	−5.4	−4.5	−4.7	−5.8	−5.6	−5.7	−5.0
** **Thymol** **	−5.8	−6.3	−5.1	−7.7	−5.9	−5.7	−5.3
** **β-Caryophyllene** **	−6.8	−6.3	−5.4	−6.6	−6.0	−7.8	−5.9
** **α-Humulene** **	−7.0	−5.5	−5.9	−6.0	−6.3	−7.6	−6.0
** **Viridoflorol** **	−7.0	−6.1	−6.2	−6.0	−6.5	−8.1	−6.5

* Protein PDB ID; 1JZQ: isoleucyl- tRNA synthetase, 1KZN: DNA gyrase, 2VEG: dihydropteroate synthase, 2ZDQ: D-alanine: D-alanine ligase, 3RAE: IV topoisomerase, 3SRW: dihydrofolate reductase, and 3UDI: penicillin-binding protein 1a. ** Ligands in red: the major volatiles in *S. officinalis* EO. Ligands in blue: the major volatiles in *T. vulgaris* EO.

## Data Availability

The data presented in this study are available in this article.

## Supplementary Materials

The following are available online at https://www.mdpi.com/article/10.3390/antibiotics11101317/s1, File S1: Molecular docking results in pdb format, ligands in sdf and mol2 formats, and receptors in pdb format.
